# The Synergetic Coupling among the Cellular Antioxidants Glutathione Peroxidase/Peroxiredoxin and Other Antioxidants and its Effect on the Concentration of H_2_O_2_

**DOI:** 10.1038/srep13620

**Published:** 2015-09-01

**Authors:** Hamid Molavian, Ali Madani Tonekaboni, Mohammad Kohandel, Sivabal Sivaloganathan

**Affiliations:** 1 Department of Applied Mathematics, University of Waterloo, Waterloo, Ontario, N2L 3G1, Canada; 2 Center for Mathematical Medicine, Fields Institute for Research in Mathematical Sciences, Toronto, Ontario M5T 3J1, Canada

## Abstract

Glutathione peroxidase (GPx), peroxiredoxin (Prx), and catalase are the major antioxidants at the cellular level and protect cell compartments against hydrogen peroxide (H_2_O_2_). In addition, they affect cellular processes such as cell signaling by modulating H_2_O_2_. In this paper we demonstrate that there is a synergetic coupling between GPxs, Prxs themselves and also with other antioxidants when the GPxs and Prxs are not in their saturated reduced form. This is due to a change in the activity of glutathione peroxidases and peroxiredoxins as a result of a change in the concentrations of other antioxidants. The strength of this synergy depends on the reaction rates and the concentration of these antioxidants. We use a perturbative method to calculate the concentration of H_2_O_2_ as function of the production rate of H_2_O_2_ and the concentration of various antioxidants. This derivation shows clearly why antioxidants behave in a correlated manner and why any change in the activity of one of them translates to a change in the activity of other antioxidants. Our results show that an increase in the activity of GPxs or Prxs might not be due to a genetic switch but due to an increase in the activity of other antioxidants.

Living organisms are constantly exposed to reactive oxygen species both intracellularly and extracellulary^1^,^2^,^3^. Hence they have developed antioxidant machinery to protect themselves against the damaging effects of reactive oxygen species^4^,^5^,^6^,^7^. Antioxidants play an important role in cellular homeostasis and the development of some of them in early life demonstrates the key role of antioxidants in cells^8^. That is why antioxidants are a key part of healthy cells and their involvement is crucial in the initiation and progress of diseases such as cancer^9^,^10^. The major players in the cellular antioxidants include the glutathione peroxidase family, the peroxiredoxin family and catalase^8^,^11^,^12^. Different cell types might have different concentrations of these antioxidants or they may be deficient in some, thus the dominant antioxidants might change from one cell type to another^8^,^11^,^12^. In addition, these antioxidants are not uniformly distributed in cells and they may exist only in one cell compartment such as catalase, which is confined to the peroxisome.

The Glutathione peroxidase family is one of the major antioxidants with eight known members^11^. Different cell types might only have a few of these members and GPxs are not uniformly distributed throughout the cells and may be localized in some specific cell compartments^11^. For instance GPx-1, which is the most abundant one in this family, is found mostly in the cytosol and in the matrix part of the mitochondria^11^,^13^, GPx-2 and GPx-3 are respectively observed in some types of endothelial cells and in the extracellular matrix^11^,^14^, GPx-4 mostly protects the biomembrane and the liposomes^11^,^15^,^16^ and, has mitochondrial, cytosolic and nucleic forms, and finally GPx-5 and GPx-6 are respectively expressed in the epididymis and the olfactory epithelium^11^. GPxs exist in reduced and oxidized forms and the oxidized form needs an intermediate reductase such as glutathione to revert to the reduced form^11^. Therefore the catalytic kinetics of this family is different from catalase.

Peroxiredoxins are among the most abundant proteins in cells with six members^8^,^17^,^18^. Prxs are mostly located in the cytosol, however they can be found in other cell compartments such as the peroxisomes and mitochondria. Similar to GPxs, Prxs exist in both reduced and oxidized forms, however, they mostly use thioredoxin (Trx) or other disulfide oxidoreductases to revert back to their reduced forms. Prxs have three distinct types of which, for the typical 2-Cys and atypical 2-Cys, Prx is not conserved; however for the 1-Cys type, Prx is conserved^8^.

Catalase, is the other cellular antioxidant which plays a key role in the detoxification of H_2_O_2_^19^. Catalase is predominantly located in the peroxisomes and, as opposed to Prxs and GPxs, does not require any reductase to continue its detoxification process^5^,^14^.

These antioxidants use their own channels to contribute to the cellular defense system against H_2_O_2_, and it appears that they behave independently. The only common factor among them is the sources of reductase which are predominantly GSH and Trx. In this work, we consider the major antioxidant system of cells and show that, in fact, there is synergy among all the members of GPx and Prx families and also between GPxs and Prxs members and other antioxidants. Furthermore, the activities of GPxs and Prxs are modulated by any other antioxidants in the cells. This phenomenon is a property of the interactions of the antioxidants (which have an oxidized and reduced form) with a reductase which converts their oxidized form to the reduced form. For instance the Prx and GPx families function under these conditions. We apply a perturbative method to the synergy of this antioxidant system and derive an analytical solution for the concentration of H_2_O_2_ and the reduced forms of Gpxs and Prxs. These solutions provide an easy way to calculate any effect in the change of antioxidants into the concentrations of H_2_O_2_ and antioxidants and also directly show the existence of the synergy among GPxs and Prxs and other antioxidants.

## Results

In the model section we obtained the expression for the concentration of reduced form of GPxs and Prxs. Interestingly the concentration of reduced forms of GPxs and Prxs depend on the concentration of other antioxidants and by increasing the concentration of one of the other antioxidants the concentrations of the reduced forms of GPxs and Prxs increase. GPxs and Prxs activity depends on concentration of the reduced form of GPxs and Prxs, hence by increasing the concentration of other antioxidants, GPxs and Prxs activity increases. This increase in the GPxs and Prxs activity means that there is a synergetic coupling among GPxs, Prxs and other antioxidants. The derived formula provides a unique way for predicting the concentration of H_2_O_2_ and the activity of GPxs and Prxs and how a change in any of the antioxidants affects the overall rearrangement of antioxidant activity.

In this section we use numerical methods to exactly solve the kinetic equations which govern the detoxification of H_2_O_2_ in order to investigate the existence of coupling between antioxidants and how this could modulate GPxs and Prxs activities. Since discussing all of the antioxidants together only obfuscates the problem we focus on special cases in which the activity of one antioxidant varies with a change of concentration in another, but hold the concentrations of all other antioxidants constant. Two of the interesting cases we discuss in this paper are the coupling between GPx1, Prx3 and Prx2. These antioxidants are three major antioxidants in the mitochondria and their activity plays a key role in some signaling processes, and also in the detoxification of H_2_O_2_ and protection of mDNA.

**Figure 1:**
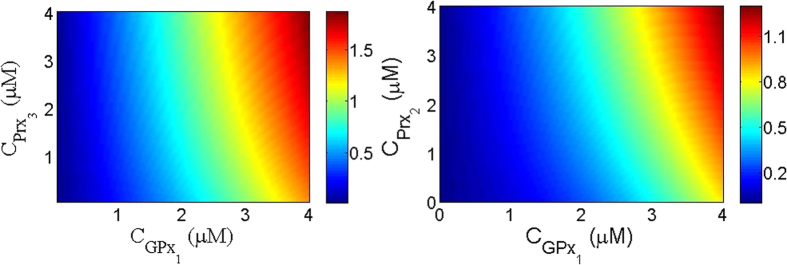
Synergetic behavior between GPX1 and Prx3/Prx2. (**a**) Prx3 activity as functions of the concentration of GPx1 and Prx3 for 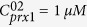. (**b**) Prx3 activity as functions of the concentration of GPx1 and Prx3 for 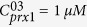. In these figures 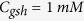, 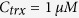 and 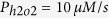. Notice to the change of Prx3 and Prx2 activity when the concentration of GPx1 changes in their corresponding figures.

To understand if there is any correlation between these antioxidants we investigate the change in the concentration of the reduced form of Prx3, as a function of GPx1. To be crystal clear, in our paper, the activity of each enzyme is associated with the concentration of the reduced form of that enzyme. In Fig. 1a we plot the concentration of the reduced form of Prx3 as a functions of Prx3 and GPx1. Interestingly, we observe that by changing the concentration of GPx1, Prx3 activity changes. Hence changing Gpx1 concentration not only changes the concentration of H_2_O_2_ but also changes Prx3 activity. This can result in a higher decrease in the concentration levels of H_2_O_2_ by Prx3. Therefore there is a synergetic coupling between GPx1 and Prx3 which amplify the Prx3 activity. In Fig. 1b we plot Prx2 activity as a function of the concentration of Prx2 and GPx1. Again, we observe the same phenomenon as for Prx3, namely that by increasing the concentration of GPx1, Prx2 activity increases and we conclude that there is a synergetic coupling between Prx2 and GPx1.

**Figure 2:**
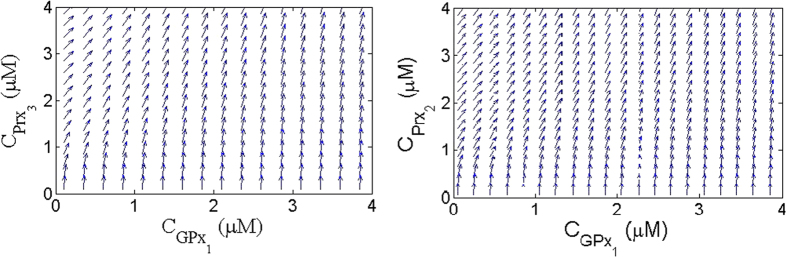
Synergetic dominant regions. (**a**) The rate of change in the Prx3 activity by changing the concentration of GPx1 and Prx3 
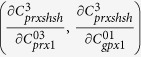
 for 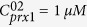 (**b**) The rate of change in Prx2 activity by changing the concentration of GPx1 and Prx2 
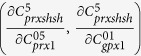
 for 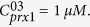 In these plots the concentrations of other species are 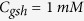, 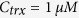 and 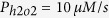.

To find out in which regions the effect of this synergetic coupling to GPx1 is more significant than the effects of Prx3 or Prx2 by themselves, we evaluate the rate of change in Prx3 and Prx2 activity as functions of the concentration of GPx1 and Prx3 and Prx2 respectively while keeping the concentration of Prx2 and Prx3 respectively constant. Mathematically for Prx3/GPx1 coupling, we calculate 
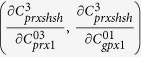
 while keeping the concentration of Prx2 constant. If the rate of change of Prx3 activity is dependent more on the concentration of GPx1, then the vectors will be more tilted towards the GPx1 axis. However, if the rate of change in Prx3 activity is more dependent on Prx3 then we would expect to observe vectors more tilted towards the Prx3 axis. In Fig. 2a we plot the rate of change in Prx3 as a function of the concentration of GPx1 and Prx3. We observe that at low concentrations of Prx3, the vectors have a big vertical component and by increasing the concentration of Prx3 they started tilting towards the vertical axis. The reason for this behavior is the competition between the increases in the activity of Prx3 by synergy verses the increase in the activity of Prx3 because of the increase in the concentration of Prx3. At low concentration of Prx3, Prx3 is dominantly in the oxidized form. Hence adding Prx3 to the system directly increases the concentration of reduced form of Prx3. However, adding GPx1 only converts a small fraction of Prx3 from oxidized form to the reduced form via synergy. In opposite, for high concentration of Prx3, the amount of oxidized form of Prx3 is already high and adding Prx3 makes a small change in the total concentration of reduced form of Prx3. In this case adding GPx1 could convert the same amount of oxidized Prx3 to the reduced form and compete with Prx3. That is why we observe that at high concentration of Prx3 the vectors are more tilted towards the Gpx1 axis and the effect of synergy becomes more predominant. In Fig. 2b we plot the rate of change in the Prx2 activity as a function of the concentrations of GPx1 and Prx2 for a given concentration of Prx3. Similar to the case of GPx1/Prx3, we observe that the synergetic coupling become important at high concentration of Prx2 and as the concentration of GPx1 increases this becomes smaller. Interestingly, in both Prx3 and Prx2 we observe that the rate of change of Prx3 or Prx2 activity with respect to the concentration of Prx3 or Prx2 shows a minimum. This minimum corresponds to the transition between an increase in Prx3 or Prx2 activity as a result of increase in the concentration of Prx3 or Prx2, respectively, or synergy. In Supplementary information we plot the rate of change of the concentration of Prx3 activity with respect to the concentration of Prx3 as a function of Prx3 to show this minimum.

**Figure 3:**
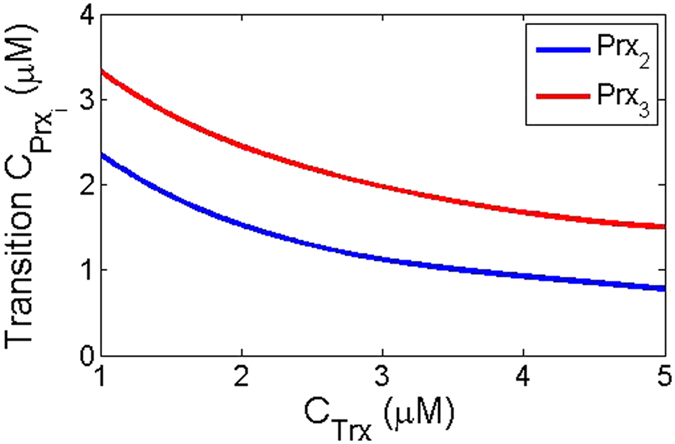
Transition point between the synergy dominant region to the normal region for (a) Prx3 and (b) Prx2 as function of the concentration of Trx.

To clarify the discussion in the previous paragraph we plot the transition from the synergy dominate region to the normal region (discussed in the above paragraph) as a function of Trx in Fig. 3. This plot shows that by increasing the concentration of Trx the transition point of both Prx3 and Prx2 is shifted towards lower Prx3 and Prx2 values, respectively. However, for Prx2 this change is very slow at low concentration of Trx. This decrease in the transition concentration of Prx3 and Prx2 is due to the increase in the reduced form of Prx3 and Prx2 which cause the transition point is shifted to lower values of Prx3 or Prx2.

**Figure 4:**
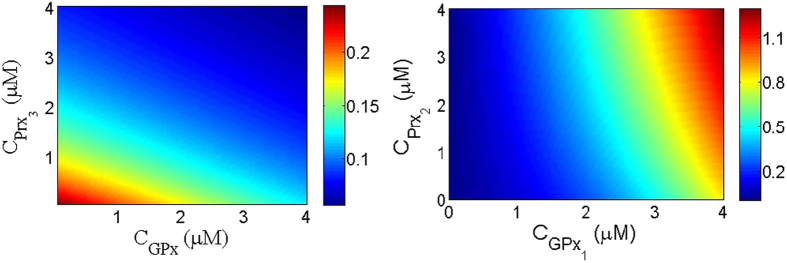
Mechanism of the synergetic behavior based on H_2_O_2_ level. (**a**) Concentration of H_2_O_2_ as functions of the concentration of Prx3 and GPx1 for 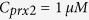. (**b**) The concentration of H_2_O_2_ as functions of the concentration of Prx2 and GPx1 for 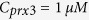. In these plots the concentrations of other species are 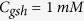, 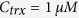 and 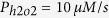.

To understand these synergetic behaviors, in Fig. 4 we plot the concentration of H_2_O_2_ as a function of the concentration of GPx1 and Prx3 or Prx2 corresponding to the presented results in Figs 1 and 2. By increasing the concentration of GPx1 or Prx3 or, Prx2 the concentration of H_2_O_2_ decreases, however, the rate of this decrease depends on the types of antioxidants. For instance, GPx1 has a higher capacity to detoxify H_2_O_2_ as compared to Prx3 and Prx2. It should be noted that the decrease in the concentration of H_2_O_2_ corresponds to the increase in the activity of Prx3 and Prx2. This explains the reason behind the observed synergetic coupling between GPx1 and Prx3/Prx2. In fact by increasing the concentration of GPx1 the concentration of H_2_O_2_ decreases. This decrease in the concentration of H_2_O_2_ converts the oxidized form of Prx3 and Prx2 to the reduced forms and increases the Prx3, Prx2 activity.

## Discussion

Observation of the synergetic coupling among the major antioxidants glutathione peroxidase family and peroxiredoxin family is an interesting and fundamental phenomenon which impacts our understanding of the way these antioxidants regulate H_2_O_2_. It shows that antioxidants may not be working as individual entities to detoxify H_2_O_2_ but that they act in harmony and in a coordinated fashion to protect cells against oxidants. Moreover, this coordinated manner of regulating H_2_O_2_ levels permits more intricate control of H_2_O_2_ concentration levels, which is critical for one of the roles of H_2_O_2_, as a cell signaling species.

The source of the observed synergy is the coupling of these antioxidants to the same substrate, namely H_2_O_2_. When the production of this substrate is high, the conversion of the oxidized forms of GPxs or Prxs to their reduced forms cannot match the rate of conversion of their reduced forms to oxidized forms. Hence, a portion of the GPxs or Prxs remain in their oxidized form. An increase in the concentration of each antioxidant would decrease the concentration of H_2_O_2_ and as a result increase the concentration of the reduced form of Gpxs and Prxs which would lead to an increase in their activity. This increase in the activity of GPxs and Prxs through an increase in the concentration of other antioxidants, translates into the synergetic behavior in these systems. In other words, at low concentrations of H_2_O_2_ all Prxs and GPxs are completely in their reduced forms, hence there is no synergy among them. However at high concentrations of H_2_O_2_, which could correspond to a high production rate of H_2_O_2_ or low concentration of antioxidants, Prxs and GPxs are not in their fully oxidized form and any variation in the concentration of GPxs could increase the activity of Prxs. We should comment that the synergy between these antioxidants could be small because of the reaction rates of Gpxi and Prxi. For example our calculation shows that the synergy is small between Prx5 and Gpx1 for all the concentration range of these antioxidants.

The observed synergy is not limited to GPxs and Prxs and other agents, which are antioxidants could reduce H_2_O_2_ at the cellular level, and also have a synergetic coupling with the GPxs and Prxs. To include these terms the reaction rates of these antioxidants with H_2_O_2_ need to be added into the current formalism.

In our calculations we neglected the spatial distribution of all involved species. Although this approximation provides a good description for phenomena that do not depend on the spatial distribution of H_2_O_2_ such as the average concentration of H_2_O_2_, in some cases a spatio-temporal model may be necessary. For example if one is interested in investigating the cell signaling which affects a receptor on the cell membrane or the distribution of H_2_O_2_ around the peroxisome, then one needs to consider the spatial distribution of H_2_O_2_. However, because of the fast diffusion of H_2_O_2_ we would not expect inhomogeneity, except for parts of the cells which are close to the sources of H_2_O_2_ or membranes. We are currently developing a model which takes into account the spatial distribution of H_2_O_2_ and other antioxidants.

In conclusion, we have developed a theoretical framework to investigate the detoxification of H_2_O_2_ by antioxidants to see if there is any coupling among the antioxidants. By using a perturbative method we have analytically derived the concentration of H_2_O_2_ as a function of the concentration of other antioxidants and of the production rate of H_2_O_2_. Using the derived concentration of H_2_O_2_ we have shown that the concentrations of the oxidized forms of GPxs and Prxs depend on the concentrations of other antioxidants. This shows that at high concentrations of H_2_O_2_ in which GPxs and Prxs are not in their saturated reduced forms any change in the concentrations of any antioxidants would modulate the reduced form of GPxs and Prxs. This proves that there is synergetic behavior between antioxidants and GPxs and Prxs. By using a numerical method we have solved the detoxifying equations for GPx1, Prx3 and Prx2 to show that this synergetic behavior does, in fact, exist among these antioxidants. We also demonstrated that there are regions in which this synergetic behavior plays a dominant role. Our results reveal a new way of understanding and thinking about the behavior of the antioxidant system in cells and may help in developing drugs to either protect normal cells or eliminate the abnormal and malignant cells.

## Methods

In our model we consider a single cell with GPxs, Prxs, and catalase as the dominant antioxidants and we assume that there is a production of H_2_O_2_ inside the cell with a given rate. We find a quasi-steady state in which the concentrations of all involved species remain constant over the relevant time scale. Because a cell has dimensions on the micrometer scale and the diffusion constant of H_2_O_2_ is on the order of 1000 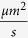 the detoxification of each of the antioxidants is not limited by H_2_O_2_ accessibility. Therefore we assume that each antioxidant detoxifies H_2_O_2_ based on its detoxifying potential although the antioxidant may be confined to a special compartment such as the peroxisome. Hence, we ignore spatial dependency in our model, and for antioxidants (which are confined to a compartment) we include the volume ratio of the compartment to the whole cell in order to capture some of the spatial effects. Besides, since the concentration of GSH is much higher than the concentration of GPxs, the change in the concentration of GSH is negligible and we assume that it remains constant on the relevant time scale.

The chemical reactions governing the detoxification of H_2_O_2_ by catalase, Prxs and GPxs are as follow,

Catalase:


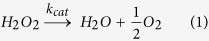


GPx:


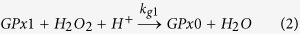



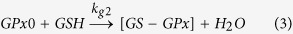



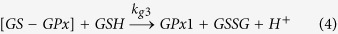


Prx (typical 2-Cys and atypical 2-Cys):


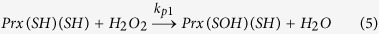



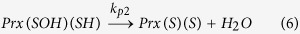



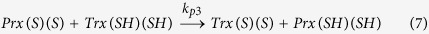



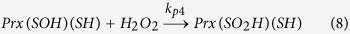


Prx (1-Cys):


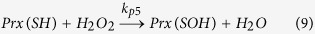



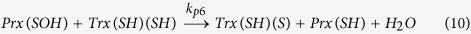


where GPX1 and GPX0 are respectively the reduced and oxidized form of GPx-1, GSSG is glutathione disulphide, GS-GPx is the glutathione-enzyme complex, Prx(SH)(SH), Prx(SOH)(SH) are respectively the reduced and oxidized form typical 2-Cys and atypical 2-Cys of Prx, Prx(SH), Prx(SOH) are respectively the reduced and oxidized form typical 1-Cys of Prx, Trx(SH)(SH), Trx(S)(S) are respectively the reduced and oxidized form of thioredoxin and Prx(SO_2_H)(SH) is the hyperoxidized form of Prx. A List of the reaction Rates are given in Supplementary information. Since the typical 2-Cys Prxs and the atypical 2-Cys Prxs function in the same way, in the above reactions we do not differentiate them.

We use the chemical reactions in equations (1) to (10), 10, 11, 10, 11, 10, 11, 10, 11, 41 to write the kinetics equations for detoxifying H_2_O_2_ assuming that H_2_O_2_ is produced at the rate 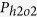 inside the cell.


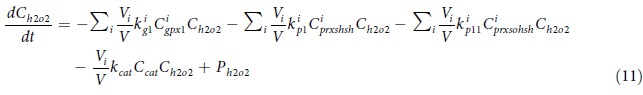



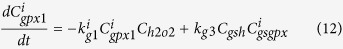



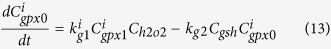



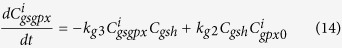



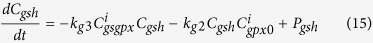



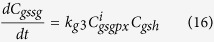



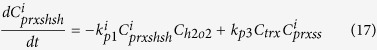



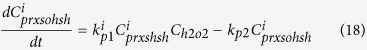



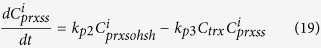



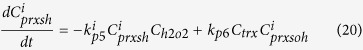



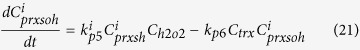


where *V*_*i*_ is the volume of compartment i which contains antioxidant i, *V* is the total volume of cell, 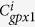, 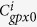, 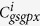, 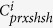, 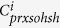, 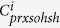, 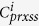, 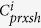, 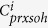, 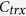, 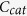, 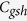, 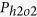, 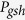 are respectively the concentration of GPX1, GPX0, GSGPX, Prx(SH)(SH), Prx(SOH)(SH), Prx(S)(S), 1-Cys Prx(SH), 1-Cys Prx(SOH), Trx, catalase, glutathione, and the production rates of H_2_O_2_ and GSH. We consider a quasi-steady-state in which the concentrations of all species remains constant over the unit of time. We also assume that H_2_O_2_ only effects each antioxidant to the first order and obtain the concentration of H_2_O_2_ in terms of other involved species (Supplementary information),


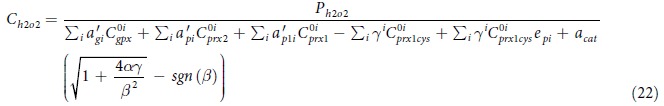


The reduced form of GPxs and Prxs can be written as,


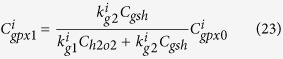



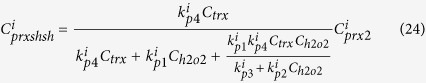



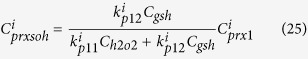


Substituting 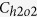 from Equation (22) into the above equations gives the reduced concentration of GPxs and Prxs as functions of the concentration of all antioxidants in the system. The approximation that H_2_O_2_ only affects the concentration of antioxidants to first order can be interpreted as the assumption that the coupling between antioxidants is mutual and higher order terms which capture to some extent the coupling between three or more terms are neglected.

## Additional Information

**How to cite this article**: Molavian, H. *et al.* The Synergetic Coupling among the Cellular Antioxidants Glutathione Peroxidase/Peroxiredoxin and Other Antioxidants and its Effect on the Concentration of H_2_O_2_. *Sci. Rep.* **5**, 13620; doi: 10.1038/srep13620 (2015).

## Supplementary Material

Supplementary Information
