# Supplementary material for: The Synergetic Coupling among the Cellular Antioxidants Glutathione Peroxidase/Peroxiredoxin and Other Antioxidants and its Effect on the Concentration of H2O2
Source: Sci Rep. 2015 Sep 1;5:13620. doi: 10.1038/srep13620 (PMC4555031; doi:10.1038/srep13620)

The Synergetic Coupling among the Cellular Antioxidants Glutathione Peroxidase/Peroxiredoxin and Other Antioxidants and its Effect on the Concentration of H_2O_2

Hamid Molavian[1*], Ali Madani Tonekaboni[1], Mohammad Kohandel[1,2*], and Sivabal Sivaloganathan [1,2]

[1] Department of Applied Mathematics, University of Waterloo, Waterloo, Ontario, N2L 3G1, Canada

[2] Center for Mathematical Medicine, Fields Institute for Research in Mathematical Sciences, Toronto, Ontario M5T 3J1, Canada

Supplement

Reaction rates, the derivation of the concentration of H_2O_2 and the rate of change of Prx3 activity as functions of the concentration of Prx3.

1. Reaction Rates

List of reaction rates:

(a)

k_{cat}	k_{g1}	k_{g2}	k_{g3}
$1.0 \times 10^7 M^{-1}s^{-1}$	$2.1 \times 10^7 M^{-1}s^{-1}$	$4 \times 10^4 M^{-1}s^{-1}$	$1 \times 10^7 M^{-1}s^{-1}$

(b)

k_{p1}	k_{p2}	k_{p3}	k_{p4}
$3.0 \times 10^7 M^{-1}s^{-1}$	$1.2 \times 10^4 M^{-1}s^{-1}$	$2.2 \times 10^6 M^{-1}s^{-1}$	$2.0 \times 10^6 M^{-1}s^{-1}$

(c)

k_{p1}	k_{p2}	k_{p3}	k_{p4}
$1.0 \times 10^8 M^{-1}s^{-1}$	$1.2 \times 10^4 s^{-1}$	$2.2 \times 10^6 M^{-1}s^{-1}$	$2.0 \times 10^6 M^{-1}s^{-1}$

List of reaction rates for (a) catalase and GPx1, (b) PRx3 and (c) PRx2 [1-4].

1. Antunes F, Salvador A, Pinto RE. (1995) PHGPx and phospholipase A2/GPx: comparative importance on the reduction of hydroperoxides in rat liver mitochondria. *Free Radic. Biol. Med.* **19**(5):669-77.

2. Trujillo, Madia, Gerardo Ferrer-Sueta, Leonor Thomson, Leopold Flohé, and Rafael Radi. "Kinetics of peroxiredoxins and their role in the decomposition of peroxynitrite." In *Peroxiredoxin Systems*, pp. 83-113. Springer Netherlands, 2007.

3. Peskin, Alexander V., et al. "Hyperoxidation of Peroxiredoxins 2 and 3 RATE CONSTANTS FOR THE REACTIONS OF THE SULFENIC ACID OF THE PEROXIDATIC CYSTEINE." Journal of Biological Chemistry 288.20 (2013): 14170-14177.

4. Manta, Bruno, et al. "The peroxidase and peroxynitrite reductase activity of human erythrocyte peroxiredoxin 2." Archives of biochemistry and biophysics 484.2 (2009): 146-154.

2. Derivation of the concentration of H_2O_2

Derivation of the concentration of hydrogen peroxide as function of the other involved parameters.

We consider a quasi-steady-state in which the concentrations of all species remain constant over the unit of time. We use Eqs. (12) to (21) to find the concentrations of reduced form of Prxs and GPxs and then substitute in Eq. (11) to get the following equation:

$$P_{h2o2} = \sum_i \frac{V_i}{V} k_{g1}^i \mathcal{E}_{gpx}^i C_{gpx}^{0i} C_{h2o2} + \sum_i \frac{V_i}{V} k_{p1}^i \mathcal{E}_{prx}^i C_{prx2cys}^{0i} C_{h2o2} + \frac{V_{cat}}{V} k_{Cat} C_{cat} C_{h2o2} + \sum_i \frac{V_i}{V} k_{p11}^i \mathcal{E}_{prx}^i C_{prx1cys}^{0i} C_{h2o2} + \sum_i \frac{V_i}{V} k_v^i C_{vr}^i C_{h2o2} \qquad (1A)$$

where C_{gpx}^{0i}, $C_{prx2cys}^{0i}$, and $C_{prx1cys}^{0i}$ are respectively the total concentration of GPx, 2-Cys Prx and 1-Cys Prx and functions $\mathcal{E}_{gpx}^i$, $\mathcal{E}_{prx2cys}^i$, and $\mathcal{E}_{prx1cys}^i$ are defined as the following,

$$\mathcal{E}_{gpx}^i = \frac{k_{g2}^i C_{gsh}}{k_{g1}^i C_{h2o2} + k_{g2}^i C_{gsh}} \qquad (2A)$$

$$\mathcal{E}_{prx2cys}^i = \frac{k_{p4}^i C_{trx}}{k_{p4}^i C_{trx} + k_{p1}^i C_{h2o2} + \frac{k_{p1}^i k_{p4}^i C_{trx} C_{h2o2}}{k_{p3}^i + k_{p2}^i C_{h2o2}}} \qquad (3A)$$

$$\mathcal{E}_{prx1cys}^i = \frac{k_{p12}^i C_{gsh}}{k_{p11}^i C_{h2o2} + k_{p12}^i C_{gsh}} \qquad (4A)$$

We could expand these functions assuming that $\frac{k_{g2}^i C_{gsh}}{k_{g1}^i C_{h2o2}}$, $\frac{k_{p4}^i C_{trx}}{k_{p2}^i C_{h2o2}}$ and $\frac{k_{p12}^i C_{gsh}}{k_{p11}^i C_{h2o2}}$ are small

$$\mathcal{E}_{gpx}^i = \frac{k_{g2}^i C_{gsh}}{k_{g1}^i C_{h2o2}} \left[1 - \frac{k_{g2}^i C_{gsh}}{k_{g1}^i C_{h2o2}}\right] \qquad (5A)$$

$$\mathcal{E}_{prx2cys}^i = \frac{k_{p4}^i C_{Trx}}{k_{p2}^i C_{h_2o_2}} \left[1 - \frac{k_{p4}^i C_{Trx}}{k_{p2}^i C_{h_2o_2}} - \frac{k_{p4}^i C_{Trx}}{k_{p3}^i + k_{p2}^i C_{h_2o_2}}\right] \qquad (6A)$$

$$\mathcal{E}_{prx1cys}^i = \frac{k_{p12}^i C_{gsh}}{k_{p11}^i C_{h2o2}} \left[1 - \frac{k_{p12}^i C_{gsh}}{k_{p11}^i C_{h2o2}}\right] \qquad (7A)$$

Substituting these relationships in Eq. 22, and simplifying the equation we get the following equation for the concentration of H_2O_2 as functions of antioxidants,

$P_{h2o2} = \left(\sum_i a'_{gi} C_{gpx}^{0i} + \sum_i a'_{pi} C_{prx2}^{0i} + \sum_i a'_{p1i} C_{prx1}^{0i} - \sum_i \gamma^i\right) C_{h2o2} - \left(\sum_i a'_{gi} b_{gi} C_{gpx}^{0i} + \sum_i a'_{pi} b_{pi} C_{prx2}^{0i} + \sum_i a'_{p1i} b_{p1i} C_{prx1cys}^{0i} - \sum_i \gamma^i C_{prx1cys}^{0i}\right) \frac{1}{C_{h2o2}} + \left(\sum_i \gamma^i C_{prx1cys}^{0i} e_{pi} + a_{vit} + a_{cat}\right) C_{h2o2}$

Where the parameters in this equation are $a'_{gi} = \frac{V_i}{V} k_{g2}^i C_{gsh}$, $a'_{pi} = \frac{V_i}{V} \frac{k_{p1}^i k_{p4}^i}{k_{p2}^i} C_{trx}$, $a'_{p1i} = \frac{V_i}{V} k_{p12}^i C_{trx}$, $b_{gi} = \frac{k_{g2}^i}{k_{g1}^i} C_{gsh}$, $b_{pi} = \frac{k_{p4}^i}{k_{p2}^i} C_{Trx}$, $e_{pi} = \frac{k_{p2}^i}{k_{p3}^i}$, $\gamma^i = \frac{V_i}{V} \frac{k_{p11}^i k_{p4}^i}{k_{p3}^i} C_{trx}$. This is a quadratic equation which could be solved analytically. The solution of this equation reads,

$$\alpha (C_{h2o2})^2 + \beta C_{h2o2} - \gamma = 0 \tag{8A}$$

$$\begin{cases} \alpha = \sum_i a'_{gi} C_{gpx}^{0i} + \sum_i a'_{pi} C_{prx2}^{0i} + \sum_i a'_{p1i} C_{prx1}^{0i} - \sum_i \gamma^i + \sum_i \gamma^i C_{prx1cys}^{0i} e_{pi} + a_{vit} + a_{cat} \\ \beta = P_{h2o2} \\ \gamma = -\sum_i a'_{gi} b_{gi} C_{gpx}^{0i} + \sum_i a'_{pi} b_{pi} C_{prx2}^{0i} + \sum_i a'_{p1i} b_{p1i} C_{prx1cys}^{0i} - \sum_i \gamma^i C_{prx1cys}^{0i} \end{cases}$$

We can solve this quadratic equation to find the concentration of H_2O_2 in terms of other involved species yielding,

$$C_{h2o2} = \frac{P_{h2o2}}{\sum_i a'_{gi} C_{gpx}^{0i} + \sum_i a'_{pi} C_{prx2}^{0i} + \sum_i a'_{p1i} C_{prx1}^{0i} - \sum_i \gamma^i C_{prx1cys}^{0i} + \sum_i \gamma^i C_{prx1cys}^{0i} e_{pi} + a_{vit} + a_{cat}} \left(\sqrt{1 + \frac{4\alpha\gamma}{\beta^2}} - sgn(\beta)\right) \tag{9A}$$

3. The rate of change of Prx3 activity as functions of the concentration of Prx3

This plot shows the transition between the synergy dominant region to the region in which the increase in the concentration of Prx3 is the result of increase in the concentration of Prx3. In Fig. the minimum of this graph is plotted as function of Trx. $C_{gsh} = 0.3\ mM$, $C_{trx} = 1\mu M$, $C_{prx1}^{02} = 1\mu M$ and $P_{h2o2} = 10\mu M/s$.

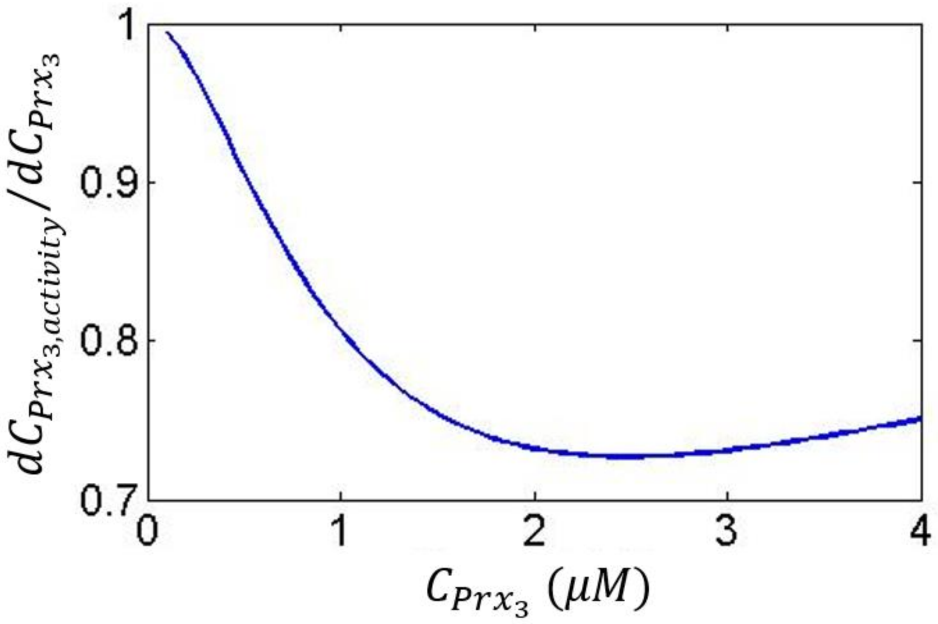

Supplement: Supplementary Information [file srep13620-s1.pdf]
